# Formative Evaluation of Participant Experience With Mobile eConsent in the App-Mediated Parkinson mPower Study: A Mixed Methods Study

**DOI:** 10.2196/mhealth.6521

**Published:** 2017-02-16

**Authors:** Megan Doerr, Amy Maguire Truong, Brian M Bot, John Wilbanks, Christine Suver, Lara M Mangravite

**Affiliations:** ^1^ Sage Bionetworks Seattle, WA United States

**Keywords:** informed consent, research ethics, mobile applications, smartphone, Parkinson disease

## Abstract

**Background:**

To fully capitalize on the promise of mobile technology to enable scalable, participant-centered research, we must develop companion self-administered electronic informed consent (eConsent) processes. As we do so, we have an ethical obligation to ensure that core tenants of informed consent—informedness, comprehension, and voluntariness—are upheld. Furthermore, we should be wary of recapitulating the pitfalls of “traditional” informed consent processes.

**Objective:**

Our objective was to describe the essential qualities of participant experience, including delineation of common and novel themes relating to informed consent, with a self-administered, smartphone-based eConsent process. We sought to identify participant responses related to informedness, comprehension, and voluntariness as well as to capture any emergent themes relating to the informed consent process in an app-mediated research study.

**Methods:**

We performed qualitative thematic analysis of participant responses to a daily general prompt collected over a 6-month period within the Parkinson mPower app. We employed a combination of *a priori* and emergent codes for our analysis. *A priori* codes focused on the core concepts of informed consent; emergent codes were derived to capture additional themes relating to self-administered consent processes. We used self-reported demographic information from the study’s baseline survey to characterize study participants and respondents.

**Results:**

During the study period, 9846 people completed the eConsent process and enrolled in the Parkinson mPower study. In total, 2758 participants submitted 7483 comments; initial categorization identified a subset of 3875 germane responses submitted by 1678 distinct participants. Respondents were more likely to self-report a Parkinson disease diagnosis (30.21% vs 11.10%), be female (28.26% vs 20.18%), be older (42.89 years vs 34.47 years), and have completed more formal education (66.23% with a 4-year college degree or more education vs 55.77%) than all the mPower participants (*P*<.001 for all values). Within our qualitative analysis, 3 conceptual domains emerged. First, consistent with fully facilitated in-person informed consent settings, we observed a broad spectrum of comprehension of core research concepts following eConsent. Second, we identified new consent themes born out of the remote mobile research setting, for example the impact of the study design on the engagement of controls and the misconstruction of the open response field as a method for responsive communication with researchers, that bear consideration for inclusion within self-administered eConsent. Finally, our findings highlighted participants’ desire to be empowered as partners.

**Conclusions:**

Our study serves as a formative evaluation of participant experience with a self-administered informed consent process via a mobile app. Areas for future investigation include direct comparison of the efficacy of self-administered eConsent with facilitated informed consent processes, exploring the potential benefits and pitfalls of smartphone user behavioral habits on participant engagement in research, and developing best practices to increase informedness, comprehension, and voluntariness via participant coengagement in the research endeavor.

## Introduction

Informed consent of participants is fundamental to the ethical practice of clinical research. Disclosure, voluntariness, and decisional capacity make up the core of valid informed consent processes [1-3]. Since the adoption of the Declaration of Helsinki in 1964, regulatory authorities in countries around the world have further codified the elements of informed consent, for example the 8 requirements described in the US Code of Federal Regulations, title 45, section 46.116 [4]. However, despite widespread consensus on the importance of informed consent and broadly on the elements included therein, ensuring research participants are truly informed remains a challenge to researchers worldwide [5].

Most of what we understand about the effectiveness of informed consent has come from studies of research participant informedness in the context of clinical trials. In their 2009 systematic review, Falagas and colleagues analyzed 30 studies of participant understanding following informed consent in clinical care and clinical trial settings between 1961 and 2006 [6]. They found that participant understanding of key elements of informed consent, such as the purpose of the treatment or study, the voluntary nature of treatment or research, the ability to withdraw, and the risks and the benefits of participation, was “adequate” (>80% of the participants having understanding graded in the study’s highest classification category) in only about half of the studies they reviewed.

In their 2014 systematic review of literature from 2006-2013 about participant informedness in clinical research, Montalvo and Larson identified risk factors for poor comprehension of informed consent topics such as low literacy, lower educational attainment, non-English speaking (for studies conducted primarily in English), and mental illness [7]. Yet, across the 27 studies reviewed, participants of diverse demographic descriptions demonstrated poor comprehension of core clinical research study concepts. For example, persistent therapeutic misconception, which is when participants do not appreciate that the defining purpose of clinical research is to produce generalizable knowledge, regardless of whether they may potentially benefit. These findings further highlight the scale of the challenge researchers face in designing effective informed consent processes.

Increasing attention has been given to improving participant understanding in clinical research. In their 2004 systematic review of the use of multimedia to improve participant informedness, Flory and colleagues found that multimedia approaches did not consistently improve understanding, a finding echoed by Ryan and colleagues in their systematic review on the same topic for the Cochrane Database in 2008 [5,8]. Instead, Flory and colleagues suggest the use of “simple” language, allowing for sufficient time to evaluate information and the opportunity to clarify misunderstandings as ways to achieve adequate participant comprehension. Others have advocated for repeated exposure to study information as a method of improving informedness [9]. Many have advocated for a multipronged approach incorporating these diverse approaches to improving informedness, including the US National Quality Forum (NQF) and the US Agency for Healthcare Research and Quality (AHRQ). Within clinical care, NQF advocates for the use of universal symbols and pictures, specifies that written informed consent documents be at a US fifth-grade reading level (age 11) or lower, and endorses “teach back” interactions to improve informedness [10].AHQR has created the Informed Consent and Authorization Toolkit for Minimal Risk Research, which incorporates “plain language” explanations with audiovisual reinforcement of key concepts (eg, pictures) coupled with “teach back” interactions [11]. Efforts to enact these suggestions have been hampered by both the time and technology required for implementation.

Mobile platforms hold promise for improving informed consent as they are enabled with visual, auditory, and tactile modes of information presentation facilitating myriad modalities of interaction, for example, listening and watching video, navigating an interactive decision tree. They also offer a scalable and customizable approach to informed consent interaction to researchers. These technologies are well suited for providing just-in-time information and facilitating self-paced learning, allowing for repeated self-directed exploration of consent topics by prospective participants.

In addition to their potential as a facilitator of informed consent processes, smartphones are enticing research tools. Smartphones are increasingly ubiquitous, now owned by 58% of US adults, including 47% of those with a household income of less than US $30,000, potentially democratizing access to research participation [12]. Rich in sensors, from gyroscopes to temperature sensitive touch screens, and enabled to collect information that is both granular and continuous, smartphones offer tremendous promise for the monitoring and assessment of human health. Furthermore, smartphones are designed to be secure, with encryption and identity protection features essential to the ethical conduct of research. Unsurprisingly, by offering a dynamic, customizable, and responsive platform for engaging participants in research and enabling rapidly scalable, longitudinal investigations, these devices are being heralded as a potential boon to human health researchers [13].

To facilitate the promise of smartphones for research, Sage Bionetworks has developed a scalable, self-guided eConsent process incorporating many of the suggested elements and approaches for improving participant comprehension described previously [14]. Here we present a mixed methods investigation of participant reaction to an implementation of this eConsent within the Parkinson mPower study, an app-based, entirely remote research study focused on tracking within-day fluctuations in certain Parkinson disease symptoms. In this new research setting, is participant engagement fulfilled? Are the challenges with traditional informed consent recapitulated? Do participants raise novel concerns or identify opportunities within the informed consent process afforded by mobile platform based studies? Utilizing open-response feedback solicited by a daily general prompt from an adult population self-reporting having Parkinson disease or not, our analysis serves as a formative evaluation of the diversity of participant experience with independent eConsent and identifies themes for further evaluation.

## Methods

We assessed a convenience sample of participant reaction to the eConsent implementation within the Parkinson mPower mobile study using a mixed methods approach.

To enroll in Parkinson mPower, prospective participants download the free and openly available Parkinson mPower app from the Apple App Store; download requires an iPhone model 4s or a more advanced version. The study received attention in the popular press when opened, including at the annual Apple product launch [15]. Several Parkinson advocacy groups also publicized the study, including the Michael J Fox Foundation [16]. Therefore prospective participants may have learned about the study from any number of public sources; there was no direct recruitment of prospective participants. Parkinson mPower is an open study; after download, prospective participants attest to meeting the study’s inclusion criteria (age 18 or older, US residents, and are comfortable with reading and writing in English) and self-administer the study’s eConsent process. The development of Sage’s eConsent, including formatting and use of icons and animations, has been previously described [14]; topics addressed in the mPower eConsent are summarized in Table 1.

**Table 1:** mPower electronic informed consent (eConsent) content.

Topic	Points addressed
Welcome	Orientation to topics to be covered in eConsent
We’ll test your understanding	There will be a quiz before enrollment
	Contact information in case you have questions
Activities	Overview of study activities
	You can skip if do not want to answer or complete
Sensor data	With your permission, study will gather data from wearable fitness device or HealthKit
	Will not access other applications, photos, contacts, text, or email
Data processing	Coding of study data
	Combination of data with that of other participants
	Data will not be sold, rented, or leased
Data protection	Coding and encryption
Data transfer and use	Transfer to US-based analysis platform
	With your permission, your data can be shared with researchers worldwide
Time needed	Time needed to perform study activities
	Notification options
Study surveys	Overview of topics to be covered in surveys
	Reminder that all questions are optional
Study activities	More detail about study tasks
Withdrawal	Participation is voluntary
	Withdrawal procedures and contact information
	Data persistence after withdrawal
Issues to consider (screen 1)	Not a treatment study
	Do not do anything that makes you uncomfortable
	If others see study notifications on your phone, they may realize you are enrolled
Issues to consider (screen 2)	Possible emotional impacts of participation
	Risks that are not known at this time will be disclosed as they are identified
Risk to privacy	Separation of personally identifying information from coded data
	Who will have access to personally identifying information
	Risks of cross border transfer
Sharing options	Participant designates if they would like their study data shared only with Sage and its research partners, or broadly with qualified researchers worldwide

Following the eConsent process, prospective participants must pass a 5-question summative evaluation before being allowed to enroll in the study (Multimedia Appendix 1); those not receiving a perfect score are redirected to the beginning of the eConsent for review. There is no limit to the number of times prospective participants can review the study information within the eConsent process and attempt the quiz. Following the quiz, participants view the long form consent document, sign this document, and are emailed their signed form. From that email, participants confirm their email address and are enrolled in the study. Ethical oversight of the study was provided by the Western Institutional Review Board (WIRB #20141369). 

We performed qualitative analysis on free-text comments submitted by enrolled participants to the daily study prompt “In what ways would you improve or change mPower?” during the first 6 months following the release of the Parkinson mPower app (March 9 to September 9, 2015). Participants providing comments included in this analysis may have been enrolled for the entire study period or for some portion thereof. The open response field does not have a character limit; participants were free to skip the prompt and remain enrolled in the mPower study. This feedback was decoupled from study data collected from the core research activities and surveys.

During the study period, 9846 people completed the eConsent process and joined Parkinson mPower. Of those enrolled, 2758 (28.01%, 2758/9846) participants submitted 1 or more responses to the daily open prompt, for a total of 7483 open-responses. Initial categorization excluded 3608 responses (48.22% of total responses). Excluded responses were explicitly not informative (eg, “No comment,” “N/A,” “idk [I don’t know]”), broadly nonspecific in content (eg, emoticons, “Done,” “Good”) *,* or unable to be interpreted (eg, “Yyg,” “Buhv”). Tech or bug reports (eg, frozen screen, button not working, prompt not audible), which were monitored, categorized, and addressed in real time, were also excluded.

We employed a combination of *a priori* and emergent codes to analyze the remaining 3875 responses (51.78% of total responses) submitted by 1678 distinct participants (17.04% of total participants). We used the 8 requirements of informed consent listed in the US Common Rule as *a priori* codes [4]. Emergent codes, for example, “the role of the control participant,” were derived as we identified novel or recurrent response content following first-pass examination of all responses submitted. Following initial development by the research team, the code book was iteratively reviewed and refined by a primary coder and an independent coder using a subsample of responses until a finalized code book was agreed upon.

Using the finalized code book, 1 or more codes was assigned to each response. The primary coder coded all responses; the independent coder verified the reliability of code assignment through examination of a random subsample of 775 (20.00%) of responses. Presumed participant typographical errors were reviewed and discrepancies resolved by consensus. We extracted findings within each code, categorized these findings into broader themes, and cross-compared. After reaching consensus on the number and spectrum of refined themes, we again examined responses to ensure thematic consistency, completeness, and robustness of the themes identified.

Participants optionally completed a demographic survey shortly after enrollment and self-report diagnosis of Parkinson disease or not, age, sex, and educational attainment among other variables [17]. We employed descriptive statistics to characterize group-level demographic information available about all those submitting responses, as well as subgroup description of those submitting coded responses, as compared with the totality of enrolled mPower participants.

## Results

### Sample Characteristics

We received 7483 responses from 2758 of 9846 enrolled mPower participants. Among the 28.01% of mPower participants who submitted any response to the open prompt (“responders”), 79.30% (2187) submitted 1 or 2 responses (mean 2.71, median 1, range 1-151 responses per respondent).

Participants were presented an additional optional demographic survey following enrollment and self-report being persons with Parkinson disease (PWPD) or controls; those declining to respond were included as controls. The mPower study data collection has been previously described [17]. Among responders, 2672 (96.88%) additionally answered at least one question within the demographic survey. 1895 (71.92%) responders self-identified as persons without PD (control) and 740 (28.08%) self-identified as persons with PWPD. Responders who provided demographic information were significantly more likely to be PWPD, female, older, and have completed more formal education as compared with the entire pool of mPower participants (Table 2).

Through initial categorization, we identified 3875 responses for coding submitted by 1697 participants, 1171 (69.00%, 1171/1697) of whom self-identified as controls and 507 (29.88%, 507/1697) of whom as PWPD (19, 1.12% did not respond). These germane responses varied in length from 1 to 388 words (mean 21.93 words, median 16.00 words) and 6 to 2006 characters (mean 116.80 characters, median 84.00 characters). Multiple codes were assigned to 623 responses (16.07% of germane responses) further reflecting the richness of feedback received. Again, responders submitting germane responses were more likely to be PWPD, female, older, and have completed more formal education as compared with all mPower participants (Table 2).

**Table 2:** Demographic characteristics of participants completing one or more demographic survey questions: all mPower participants versus responders and all mPower participants versus germane responders.

Demographic characteristics	All mPower participants	Responders^a^	Germane responders^a^
	n=9846	n=2635	n=1678
Self-reported diagnosis, n (%) Parkinson disease	1414 (14.36)	740 (28.08)	507 (30.21)
	n=9986	n=2662	n=1695
Sex, n (%) female	2152 (21.55)	686 (25.77)	479 (28.26)
Age, mean years	35.90	41.75	42.89
	n=10,048	n=2672	n=1697
Education, n (%) ≥ 4-year college degree	5781 (57.53)	1620 (60.63)	1124 (66.23)

^a^*P* value versus all mPower participants; *P*<.001.

### Qualitative Analysis of Open-Response Data

#### Participant Comprehension

We selected *a priori* codes to target participant comprehension within the open-response data as the core purpose of the eConsent is to facilitate participant comprehension. Responses echoed many of the challenges reported in “traditional” informed consent processes; however, because of our methodological approach, we were not able to provide metrics for comparison with qualitative studies of participant comprehension in traditional settings [6].

##### Purpose

Some responders made statements clearly illustrating their understanding of the core purposes of the study:

I am encouraged to be on cutting edge of using technology to improve health for people...We can truly learn about disease through this better knowledge of symptoms in real time.

Participant d8ca

I feel that this app on this iPhone is fantastic because it allows the average person to get involved with PD research, instead of perhaps 3000 to 5000 research subject(s) worldwide. [Participant 00de]

However, others expressed misunderstandings, as in this example of therapeutic misconception, that is, that the app may have therapeutic benefit for Parkinson disease, whereas it was designed to track symptoms:

How do I know that this kind of mental activities and exercises work for Parkinson’s disease? All this activities help with that?

Respondent 0244

Responders expressed variable appreciation of the link between the purpose of the study and study procedures. For example, here the responder does not connect one of the core aims of the study, to capture within-day variation in Parkinson disease symptoms, with the periodicity of study activities:

...it seems like (completing study activities and surveys) two or three times a week would be an adequate measure of the change in symptoms. [Respondent 53ee]

##### Voluntary Nature of the Study and the Right to Withdraw

The right to skip activities and survey questions, as well as the right to withdraw, is highlighted throughout the eConsent process, with 2 of the 5 mPower post-eConsent comprehension quiz questions focused on this topic. Responses belied a spectrum of understanding of respondent rights as research participants to limit or stop participation. Among those fully appreciating their right to self-regulation, in addition to the autonomy expressed, we commonly noted an invested, informing communication style as if the participants viewed themselves as coinvestigators.

I am on a three day vacation. I will only be doing the memory and tapping and voice.

Respondent b383

I am not feeling well and am going to bed early. Wil(l) not finish all my activities today. [Respondent 0e8e]

However, not all participants appeared confident in their right to self-regulate their participation.

I’m going to take time off starting now, but will be back in a couple of months if that’s permissible.

Respondent d0ae

##### Risks and Benefits

The majority of statements coded in this category relate to the emotional experience of participation, a topic that is stressed as both one of the primary risks and primary benefits of participation by the eConsent process. Participants clearly comprehended this risk or benefit and were unhesitating in sharing their experience through the open-response prompt.

Respondents expressed enthusiasm, stated being happy to participate, being stimulated by being part of the research, excited, and curious. Expressions of altruism and agency were common.

I very much like participating. I feel as if I am helping to reach an overall outcome. [Respondent a88e]

I don’t have Parkinson’s but am happy to participate in the study if it helps to find help for those who have it.

Respondent c073

Negative emotional expressions were also common and ranged from boredom to frustration, stress, disappointment, and anxiety and guilt when study activities or surveys were missed.

*The memory question gets hard very quickly. It is hard on the ego...* [Respondent d9cc]

I just feel that if I forget (to complete study activities)...it’s going to make me feel even worse that I have PD.

Respondent ddb1

For both PWPD and controls, study participation prompted deep contemplation of life with Parkinson disease.

...After going through that last series of questions though I’m going of quit this program. I don’t like going through all those symptoms that I don’t have yet. I don’t want to think about what may be coming... [Respondent 2945]

This study is helping me accept the reality of my brother’s Parkinson’s diagnosis and what lies ahead for him.

Respondent 934e

We emphasized throughout the eConsent that there were no anticipated individual-level benefits expected from participating in the study, although participants would be able to track their own data, export, and share it if they chose. Respondents cited access to their own data as the primary benefit of participation and expressed frustration with any limitations to their access.

I can’t get the program to let me look back over more than a week of data. Is that my ineptness or part of the program (?) I was hoping over the years to come to see the course. [Respondent 2e3c]

The “benefit” of data tracking was often described in more nuanced terms by PWPD respondents.

I’m going to be very interested in seeing the test results over time. I think I’m losing ground and that my results are not as good now as they were when I first began.

Respondent 2b84

A handful of responses highlighted risks that were not specifically addressed in the eConsent process. Apart from battery life, these risks are not unique to mobile studies; however, they may arise more commonly in entirely remote, app-mediated studies (Table 3).

**Table 3:** Risks raised by responders not highlighted within the electronic informed consent (eConsent) process.

Finding	Example response
Feeling uncomfortable, identifiable completing study activities	*The walking exercise is a bit embarrassing around others.* [Respondent bfb3] *...Ahhhhing loudly in public causes considerable concern and consternation.* [Respondent f56a]
Apparent contradiction of treating clinician	*I can’t figure out why my gait score is low...Maybe...it is indicating something that really isn’t related to PD. I walked for my movement specialist yesterday and it looked great. He is (from a renowned hospital).* [Respondent 4419]
Battery life	*When the app is tracking the walking/gait, the phone battery drains to 50% by mid day.* [Respondent 9cb6]

##### Privacy and Confidentiality

Because of the novel approach to data capture and transfer within this app-mediated study, data handling, storage, transfer, privacy, and data confidentiality are a major focus of the eConsent. Proportionally, nearly 3 times as many PWPD commented on these topics as compared with controls (PWPD n=47, 9.27% of PWPD germane responders; controls n=37, 3.16% of control germane responders). Participants’ limited understanding of study procedures often complicated their comments about privacy and confidentiality.

iOS reported that you’re recording my location data all the time. What is the reason for that?

Respondent fab5

I am concerned about you accessing my microphone when I’m not aware of it. Make it clear that you cannot do so at any time. [Respondent 7dd8]

##### Study Procedures

Participants expressed a lack of clarity regarding study procedures. The majority of these responses focused on how to complete study activities and surveys, including activity timing and frequency. While lack of clarity on study procedures is not a unique challenge to remote, app-mediated research, participants’ approach to resolving their confusion may be. Although we listed study contact information (phone, email, and physical address) within the eConsent, written and signed consent document, and the app itself, 319 respondents submitting germane responses (19.01% of germane respondents) chose to ask questions about study procedures through the open-response prompt at least once, perhaps viewing it as a conduit for direct, immediate communication with study staff, as if texting.

Why is the study not asking me to walk carrying the phone? I’m confused.

Respondent 1e51

Additionally, artifacts of the participant’s own phone model’s impact on available study activities were misunderstood as procedural design, a challenge unique to app-mediated research.

I was wondering why I get a walking test, as a healthy participant, but my husband, who has Parkinson’s, does not get that test. [Respondent 62cb]

##### Contact With Study Staff

As previously discussed, some respondents may have felt they were “texting” the study team through the open-response field. We identified a dozen responses that contained email addresses, phone numbers, and full names in addition to numerous requests without identifying information for study staff to contact the respondent directly, as in this response:

(This particular study activity) cancels me out. Please help! Email me at (redacted) ASAP.

Respondent c6ab

##### The Definition and Role of Controls

According to the study design, both PWPD and controls were invited to complete the same study surveys and activities. The role of control participants is explicitly addressed in the eConsent and written consent, as well as in the eligibility criteria. However, myriad comments addressed the definition and role of controls, with 594 control respondents (50.73% of control germane respondents) raising questions or concerns about their role as controls and their potential impact in the study; no PWPD respondents commented on this theme.

Responses fell into 4 categories: confusion about whether controls were desired members of the study, if the study “knew” who was a control, reiteration of “healthy” status, and suggestions on how to fix the “control confusion.” Lack of clarity on the control group definition and role led to questions about the study app’s design, performance, and the overall study’s design.

The initial survey has a question that asks if I have ever been diagnosed with Parkinson’s disease: whether I answer yes or no it then asks a bunch of questions that seem to assume that I have and am being treated for Parkinson’s. It’s confusing and makes it seem like the survey is broken. [Respondent 5aa5]

(For us) participating that do not have Parkinson’s, some questions are unclear. The option to skip is available but I actually found myself checking the study requirements again just to be certain was eligible.

Respondent a7b4

### Governance Challenges in Remote Research, App-Mediated Studies

Through emergent coding, we identified informed consent process themes that may have arisen either due to the remote setting of the Parkinson mPower study, its app-mediated design, or due to both of these factors in tandem.

#### Participant Engagement

One of the dominant themes within the coded responses was participant engagement in the research ecosystem; the open-response prompt, “In what ways would you improve or change mPower,” was designed to engage participants in this way. However, we were impressed by the depth and breadth of suggestions offered and, moreover, with the ways in which these suggestions could impact informed consent concepts and processes. How to improve motivation to participate and the role of participants as research partners were at the forefront of comments.

##### Motivators

Respondents asked generally for “motivators” or “incentives” to encourage their participation. They specifically called out data access, feedback from the research team, gamification, and group or team participation as motivators to sustain their interest. There were no responses that mentioned financial incentives or transactional incentives (eg, physical “gifts”) other than data access.

I lost interest/motivation and stopped recording for a while...I think seeing my long term trends would help me stay motivated. [Respondent 5b31]

Please give feedback - are you still doing this study? I need some motivation if you want me to continue...How about a message to all the participants?

Respondent 5f9e

One suggestion is an incentive program...Incorporate tangible goals and visual reward either through points or badges. Establish goals for members so that they accomplish each task in a certain time for bonus rewards.

Respondent 8e33

Make everything a game. Make it interactive and fun. Even though kids are the ones supposed to be playing games to learn, it makes it easier for adults too. It also makes it fun and makes me want to come back to finish the task every day...even the smallest rewards are still rewards and humans thrive to be rewarded.

Respondent 59f9

At least one participant noted that in a condition for which apathy is a symptom, as in Parkinson disease, fostering motivation to participate is of particular importance.

It would probably mess up your double-blind nature or privacy but it is my experience that those of us with Parkinson’s will often show up and be more consistent when we are involved in a group rather than as individuals because of our inherent lethargy and apathy. [Respondent c1ce]

##### Citizen Science

Respondents rejoined the open-response prompt as lay scientists. Frequently responses included hypotheses about why the respondent’s own scores might be high or low, or observations about factors that might affect study performance:

Time of day affects performance irregardless (sic) of medication. Memory activity better in morning (after coffee!).

Respondent 9a05

Additionally, responders expressed their own desired purposes for the study, most commonly that the purpose should include the diagnosis and treatment of Parkinson disease.

I think it would be good to include tests that tell you if we may have Parkinson’s, so we could go to a doctor and have ourselves checked. [Respondent 4d46]

(I would like) Feedback from experts on any improvements to my medication.

Respondent 16f3

Speaking to the subtheme of alternative purposes for the study, 183 respondents (10.91% of germane respondents) asked for feedback from the study about their own study data. Proportionally, this request was 4 times more common among PWPD (n=117, 23.08% of PWPD germane respondents) as compared with controls (n=66, 5.64% of control germane respondents).

I am puzzled by the gait and balance exercise. I walk 35 steps and get a score of (number redacted). What does that mean? [Respondent f4da]

Respondents especially desired to compare their results with those of other participants, seemingly to derive greater understanding of their own disease course.

An updated (way to) compare your symptoms with others (names excluded) would be cool. As an early onset patient, I wonder how I am fairing compared with people like me.

Respondent 345a

If I could see my results compared with another would help me understbnd (sic) better how this is affecting me. [Respondent 856c]

##### Presentation of Information

Despite our sincere efforts to design the eConsent as a multimodality informed consenting process for a broad audience, respondents had suggestions for improvement. Responders made specific suggestions for refining the presentation of information throughout the eConsent, calling out the need to increasing the clarity of the eConsent process by adding detail and audiovisual materials to aid understanding, adapting the eConsent more completely for those with visual impairment, and through simplifying the language used throughout the eConsent.

If you expect people withsome high schoolto use this app you will have to simplify the vocabulary & cut out the jargon...

Respondent ebab

## Discussion

### Principal Findings

Our qualitative analysis of participant open-response feedback provides an initial description of the essential qualities of participants’ experience with a self-administered eConsent. We find our qualitative assessment of particular value not only for thematic comparison of the “lived experience” with an entirely remote mobile eConsent with existing understanding of traditional informed consent processes, but also for identifying novel and emergent themes in eConsent that may have consequences for the content contained therein.

Despite attention to presentation, content flow, and the use of icons, animations, and video as well as the volume of the information presented, we identified broad thematic consistency with gross challenges observed in in-person, fully facilitated informed consent processes. We were unable to comment on the relative degree to which participant misconception persists in self-administered eConsent as compared with traditional facilitated consent; now that we have established thematic consistency between participant experiences with the 2 modalities of consent, comparison between the approaches could be undertaken in a controlled study.

Respondents showed variable appreciation of core elements of informed participation, for example therapeutic misconception. This finding was of particular interest to our research team. Although therapeutic misconception is 1 of the most commonly discussed challenges in informed consent, researchers have previously suggested that the setting of clinical research within the academic medical center environment leads to participant conflation of research participation with clinical care [18,19]. What motifs, beyond physical setting, used both in traditional informed consent and eConsent, lead to therapeutic misconception and what approaches can be trialed to target it in remote research settings? Additional investigation is warranted.

Another stumbling block to informed and engaged participation highlighted within the open-response data was the struggle with the definition and role of the control participant. We found little discussion of this challenge within the literature, perhaps because within in-person studies, control participants are reassured frequently—albeit perhaps not consciously or deliberately—by study staff of their role and importance. It will be critical to the success of remote, app-mediated research to find balanced ways of reinforcing to control participants the requirements of their role and reaffirming their importance to research outcomes.

App-mediated research poses unique privacy and confidentiality concerns for research participants that may have implications for the content of consent. Overall, these risks were not commonly commented on by control subjects, but more than 9% of PWPD submitting responses touched on this topic. We did not find evidence in the literature of known differences with privacy and security concerns between “case” and control research participants in traditional clinical research settings. We wonder if PWPD may be more concerned about privacy and confidentiality than control participants due to their older age as compared with controls (which may engender greater skepticism of mobile technology.) Alternatively, could we observe this trend because PWPD, due to their disease status, have greater awareness and concerns about the spectrum of potential misuse of their data? Research to tease apart if the privacy and confidentiality considerations of affected populations results from rightful awareness and concern or some other source is clearly needed as the spectrum of app-mediated research studies diversifies.

Technology blurs the line between research participation and every day smartphone interaction. We attempted to design for this shift in the fundamental context of research, but were still surprised by the evidence we found of the powerful influence of habit. The risk raised by participants of being identifiable when completing study activities in public, although not unique to app-mediated research, may be exacerbated by it. For example, participants, conditioned to responding to their smartphones, may immediately move to fulfill study activities upon receiving automated notifications without pausing to contemplate if they are in an “appropriate” setting for engagement. Furthermore, the frequency of “texting” of study staff through the anonymous response field was eye opening for our research team. These “habits” have clear implications for the content addressed in the informed consent process, from highlighting the risks of identifiability to clarifying the mechanisms of study staff contact. Attention should be paid by mobile study designers to the mechanisms for study staff contact that are included in app-based studies as well as the selection and design of open-response prompts or fields. Consideration of texting or short message service (SMS)–based study contact during designated “office hours” may be a solution, although the privacy and confidentiality risks posed by texting interactions within the context of human subjects research should be assessed.

Among the dominant benefits of participation identified by respondents were the positive emotions generated by their participation including altruism and agency. At the same time, participants did not hesitate to ask for “motivators” or “incentives” to encourage their participation. The balance between intrinsic and extrinsic motivating forces in human subjects research leans away from extrinsic motivators—most commonly financial incentives—and skews heavily to intrinsic motivation as a way of avoiding the hazards of undue influence and involuntary participation [20-23]. By contrast, app “stickiness”—the ability of an app to bring its audience back time and again—is viewed as essential to successful app design [24]. As we start to recognize participants’ habitual patterns of smartphone interaction, we must guard against designs that angle toward undue influence and recognize the multifold challenges of creating app-mediated research that is engaging but not coercive, honoring the core consent principle of voluntariness in research.

One possible solution to the balance between intrinsic and extrinsic motivators is harnessing participants’ eagerness to engage as coinvestigators. One of the great promises of mHealth research is coengagement of participants and researchers. This promise is noteworthy for governance and ethics professionals as it has potential for improving participant comprehension and retention. Based on the volume and diversity of the responses we reviewed, participants are ready and able to share their insights and ideas with researchers. We plan further development of interactive study features to more actively and reciprocally partner with study participants.

### Limitations

We focused the development of our eConsent for mHealth studies on facilitating disclosure, comprehension, and voluntariness for participants independently self-administering consent for research. The eConsent has a deliberately structured format and carefully curated content designed to maximize participant understanding and engagement. Our design is openly and freely available through GitHub (GitHub, Inc). Parameters limiting the application of our work include that the eConsent prototype has been designed for use in low risk studies, with populations that have smartphones and are comfortable using them.

Within the Parkinson mPower study, respondents report having completed significantly more formal education as compared with the US general population. The study participant pool also skews strongly male as compared with the US general population. The sample of respondents is somewhat more balanced, but still skews heavily male as compared with the US general population. Germane respondents were further differentiated from the total pool of Parkinson mPower participants by reporting more formal education, having a Parkinson disease diagnosis, and being older than the total pool of participants. Based on these factors, our results may highlight the interests of PWPD, men, and those who are more highly educated over those of a more representational sample.

### Conclusions

This analysis of participant open-response feedback provides a preliminary snapshot of the consent landscape of entirely remote research administered through smartphones. While acknowledging the limitations of using general open-response feedback to address specific study questions, we identified several formative themes worthy of further consideration within informed consent in the emerging field of app-mediated research [25]. We found that, as in fully facilitated informed consent processes, ensuring participant comprehension continues to be a challenge in eConsent; now that thematic consistency has been established qualitative comparison of these 2 approaches with informed consent is warranted. We documented several study governance themes that may be exacerbated by, if not entirely unique to, app-based remote research settings, especially several ripe for inclusion in the risks and benefits of this and future similar studies. Finally, we highlighted opportunities for participant engagement that may specifically foster informedness and comprehension in remote research studies.

## Appendix

Multimedia Appendix 1

## Abbreviations

PWPD: persons with Parkinson disease
