# Supplementary figures and images for: Formative Evaluation of Participant Experience With Mobile eConsent in the App-Mediated Parkinson mPower Study: A Mixed Methods Study
Source: JMIR Mhealth Uhealth. 2017 Feb 16;5(2):e14. doi: 10.2196/mhealth.6521 (PMC5334514; doi:10.2196/mhealth.6521)

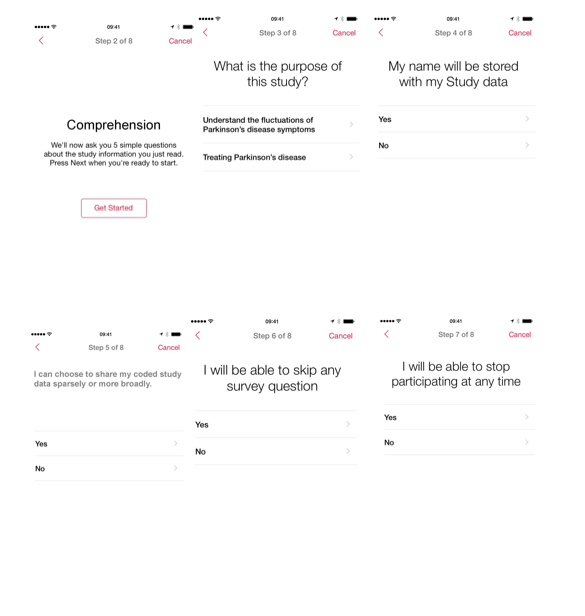

Supplement: Multimedia Appendix 1 [file mhealth_v5i2e14_app1.png]
